# Development of a Fitness Test Battery for Special Weapons and Tactics (SWAT) Operators—A Pilot Study

**DOI:** 10.3390/ijerph18157992

**Published:** 2021-07-28

**Authors:** Megan Sax van der Weyden, Christopher D. Black, Daniel Larson, Brian Rollberg, Jason A. Campbell

**Affiliations:** Department of Health and Exercise Science, University of Oklahoma, Norman, OK 73019, USAcblack@ou.edu (C.D.B.); larsondj@ou.edu (D.L.); brollberg1992@gmail.com (B.R.)

**Keywords:** tactical, fitness testing, police, anaerobic capacity, muscular endurance, aerobic capacity

## Abstract

This investigation examined relationships between a Special Weapons and Tactics-specific fitness test (SORT) and an obstacle course (OC) used for qualification in fourteen male SWAT members from three local, regional police departments. The SORT included: squat, pushup, and lunge in 60 s; pullup hold; sled drag; and Yo-Yo Intermittent Recovery Test L1. The obstacle course included: 25 m sprint (repeated); window ascent; scale under a wall; 25 m serpentine run (repeated), body drag (20 m, repeated). Pearson coefficients examined SORT and OC relationships (*p* ≤ 0.05); intraclass correlation coefficients (ICC_2,1_) assessed agreement of SORT trials. Repeated measures ANOVA evaluated differences in SORT metrics across time. Coefficients of variation (COV) examined SORT scoring consistency. The YoYo test was related to all SORT assessments (r = −0.803–0.894), except sled drag. The remaining SORT metrics were related to ≥two tests. SORT COVs ranged from 0.77–13.26% for trials 1–2 but decreased between trials 2–3 (0.95–8.97%). The OC was associated with YoYo, lunges, squats and sled drag (*r* = −0.790, −0.730, −0.766, and 0.802, respectively). No differences (*p* > 0.05) existed across SORT trials for event scores. The SORT battery appears to be a valid and reliable testing measure to assess SWAT occupational specific fitness.

## 1. Introduction

Law enforcement officers (LEO) are considered “tactical athletes” or personnel that require unique training strategies aimed at optimizing performance in physically demanding occupations, meeting objectives and overcoming various threats [1]. Due to the nature of their jobs, tactical athletes are often required to attend training academies or camps and successfully complete a variety of fitness tests before they can serve in their new occupation. These fitness tests are often composed of aerobic endurance and local muscular endurance events, using body mass as the primary form of resistance [2,3,4]. While these fitness tests, often derivatives of Cooper’s Test, may be adequate for training recruits and testing fitness maintenance in LEOs, a test that only focuses on cardiovascular endurance and local muscular endurance may be inadequate for specialist police [5].

Special Weapons and Tactics (SWAT) officers are routinely engaged in activities not typically required of LEOs such as hostage negotiations, counter-terrorism operations and large scale illicit drug apprehensions, as well as executing high risk warrants [6,7,8]. Frequently, this requires SWAT officers to sprint, vault over obstacles, maintain tactical positions for extended periods and drag casualties to safety [6,9,10]. These tasks are performed while wearing personal protective equipment (PPE) and carrying all their additional gear. This may represent a 40 kg load that SWAT operators must don [11]. Thus, the tasks SWAT officers engage in on a day-to-day basis may require muscular strength, power, speed, agility, as well as cardiovascular and local muscular endurance.

The development of a fitness battery that accurately assesses a SWAT officer’s ability to safely execute these heightened demands would seem to be of great importance. Previous research has shown that the time taken to complete a simulated tactical task increased by 7.8% when an officer was wearing a load [12]. Additionally, load carriage may lead to increased exposure to enemy fire, reduced ability to accelerate when running, and increased ratings of perceived exertion on occupational tasks [12,13]. While general LEO’s loads are approximately 10 kg, SWAT operators essential gear can weigh 22 kg with ballistic shields, battering rams and other forceable entry tools adding another 13.6 kg, 15.9 kg, and 10 kg, respectively [11]. Therefore, while Cooper’s Test may be able to adequately assess the general fitness of LEOs, it may lack sufficient scope to indicate an officer’s capacity to perform under heavy loads or a SWAT operator’s aptitude for success during high-risk callouts. Recently, it has been stated that the specific work environment and job demands of LEOs should strongly factor into the design and implementation of training and testing in these populations [14]. Failing to do so with SWAT operators may result in decreased preparation for high-intensity situations and threats to mission safety and success.

Therefore, the SWAT Operator Readiness Test (SORT) was created, following three-months of SWAT officer observation, to better model the unique physical requirements of this specialist LEO population. Containing six events, the SORT tests muscular strength, anaerobic power, mobility and agility as well as local muscular endurance and cardiovascular endurance. Additionally, the SORT requires SWAT officers to wear various loaded vests while completing the events to better model the physiological demands of PPE load carriage on operations. Therefore, the purpose of this study is twofold. First, we sought to discern the test–retest reliability of this novel test battery aimed at assessing job task preparedness among SWAT operators. Additionally, we compared the SORT and Cooper’s Test reliability for predicting an obstacle course, and thus occupational performance.

## 2. Materials and Methods

### 2.1. Subjects

A non-random sampling method was utilized as participants were recruited from three separate police departments in the local region. The convenience sample technique has been used in previous studies [10,15,16]. Our cohort was comprised of 14 male active-duty, part-time SWAT operators (age: 35.68 ± 5.82 years; height: 1.78 ± 0.08 m; body mass: 89.93 ± 12.73 kg).

### 2.2. Procedures

The research design was a test–retest design with statistical comparisons being made between two methods of physical fitness testing (SORT and Cooper’s Test) and their relationship to SWAT obstacle course outcomes. A retrospective data analysis was also performed, as data for the Cooper’s Test and obstacle course performance from the two previous years that directly preceded the current investigation were analyzed and compared to assess the strength and direction of the relationship between these two assessments. The SWAT Operators Readiness Test (SORT) battery and the obstacle course assessments were performed on different days with at least five days of recovery in-between. The tests were typically conducted between 7:00 a.m. and 12:00 p.m. Weather conditions were typical for a Midwest region of the United States in summer and were consistent (no excessive humidity or heat) across all three trials. Approval was obtained from the University’s Institutional Review Board and informed consent was received from all participants.

Participants’ height and mass were collected using a scale (Detecto, Webb City, MO, USA) before data collection. Each participant completed the SORT battery three times with no less than seven days between testing sessions. Random testing order was assigned to each participant. All tests were completed wearing typical physical training/workout attire. Due to the novelty of the test, all events were considered individually, rather than cumulatively based on a total score.

### 2.3. SWAT Operator Readiness Test (SORT)

**Figure 1:**
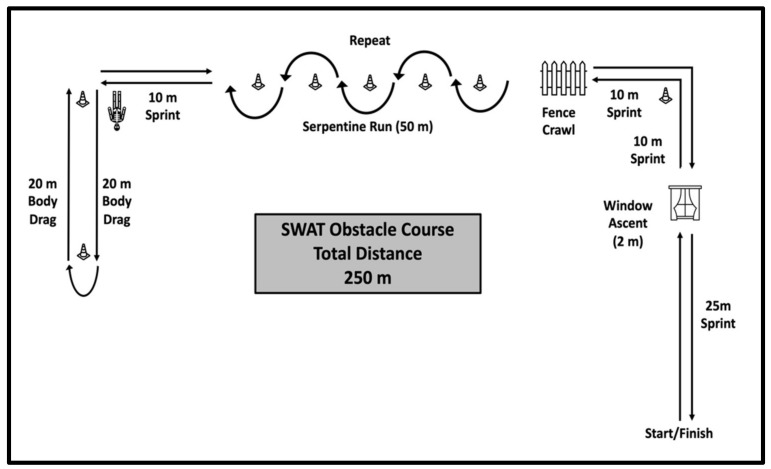
The 250 m SWAT Obstacle Course.

The SORT consists of six events: six-point weighted lunge, loaded pushup, isometric pull-up hold, loaded squat, sled drag, and Yo-Yo Intermittent Recovery Test Level 1. The weighted lunge, pushup, pull-up, and loaded squat were all performed indoors, while the sled drag, Yo-Yo intermittent test and obstacle course were completed outdoors. The Yo-Yo test and sled drag occurred on a clean, flat concrete surface. The obstacle course (Figure 1) was performed on a grass surface at the SWAT facility.

*Six-Point Weighted Lunge (WL)*: Lower body mobility and unilateral strength were assessed using a continuous lunge matrix. Operators wore a 40kg weighted vest (Mir, San Jose, CA, USA) while performing a series of lunges forward and backward. Specifically, the operator holds a PVC pipe across their shoulders to help maintain an upright position and then lunges forward with the right leg, forward with the left leg, forward at a 45-degree angle with the right leg and forward at a 45-degree angle with the left leg. Then, the operator lunges backwards in the same pattern. The total amount of repetitions completed in 60 s is recorded.

*The Loaded Push-Up (PU)*: To assess upper body “pushing” capacity, operators equipped with an 18.2 kg weighted vest (BCG/Academy, Katy, TX, USA) were asked to perform as many push-ups, with proper form, as possible in 60 s. Participants started in the “up” position, with hands shoulder-width apart and fingers facing forward. The participant was instructed to maintain a tight, straight body line. A foam block of adjustable height was placed below the chest of the participant. The operator was told to contact the foam block lightly on each repetition on the “down” portion of the movement prior to ascending to the start position. The participant received one verbal warning if the block was missed or if body position was not maintained. On the second “fault”, the test was concluded, regardless of time remaining.

*Isometric Pull-up Hold (PH)*: The pull-up hold provided a measure of upper-body pulling strength/capacity. This test, often referred to as a flexed arm hang, was continued until failure. The bar was gripped in an overhand manner, with the hands shoulder-width apart and the remainder of the body in a straight, vertical position. The test was terminated when 90 degrees flexion was broken at the elbow joint or the operator voluntarily stopped.

*The Loaded Squat (SQ)*: In the loaded squat, operators wore a 40 kg weighted vest. Each operator then completed as many squats, parallel or lower, as possible in 120 s. For each repetition, the participants had to pause for five seconds at the bottom of the squat before returning to the starting position. A PVC pipe was again placed across the operator’s shoulders and maximum repetitions were recorded.

*Weighted Sled Drag (SD)*: For time, a sled with 106 kg was pulled for 20 m across a measured flat surface marked with cones. Timing began when the front of the sled crossed the start line and ended when the front of the sled crossed the finish line. Participants donned two straps that slipped over the arms/shoulders and then faced away from the sled toward the finish line. The participant was asked to give a “ready” signal with a head nod to help ensure the timer was in sync and observing.

*Yo-Yo Intermittent Recovery Test Level 1 (YO)*: It was conducted per published protocol with a maximum number of shuttles completed recorded, and relative VO_2max_ calculated. [17].

### 2.4. Cooper’s Test

The Cooper’s Test consists of maximum push-ups and maximum sit-ups in 60 s, a 300 m timed run, and a 2.4 km timed run conducted per published protocol [5].

### 2.5. SWAT Obstacle Course

The obstacle course in the current investigation was utilized because it is a requirement for SWAT operator qualification in the current cohort and can be seen in Figure 1. Briefly, it consisted of a timed run across a 250-m distance, designed to simulate a foot pursuit that involves a 25-m sprint, climbing into a 2-m-high window, crawling under a fence, a 25-m serpentine run (2 times), crawling under a fence, climbing out of a 2-m high window, a 20-m body drag (2 directions for 40 m total) and a 25-m sprint to the finish in the fastest time possible. All participants completed the obstacle course as part of their normally scheduled duty schedule via SWAT Command at least 2 weeks prior to the completion of any SORT battery testing. This decision was made to reduce any risk of injury that may jeopardize the officers ability to work as a result of residual fatigue induced by the SORT battery testing.

### 2.6. Statistical Analysis

All statistical analysis was conducted using the Statistical Package for the Social Sciences (SPSS, Version 27 (IBM, New York, NY, USA). All data are reported as means ± SD unless otherwise noted. Statistical significance was set at *p* ≤ 0.05. Descriptive statistics were calculated for each test parameter. Bivariate correlations were calculated to compare SORT battery events to one another. Intraclass correlation coefficient (ICC) estimates and their 95% confidence intervals (95% CI) were calculated based on an absolute agreement, two-way mixed-effects model using the single measures value to assess the test–retest reliability of each SORT event across trials at each time point. The following criteria were used to classify the ICCs: <0.5 = poor reliability, 0.5–0.75 = moderate reliability, 0.75–0.9 = good reliability and >0.90 = excellent reliability Coefficients of Variation (COV) were calculated across all three trials, between trials 1 and 2, and between trials 2 and 3, to determine variability in score dispersion. Bland–Altman plots were created to test agreement between trial 2 and trial 3 and to assess bias across the normal range of scores for each of the SORT battery tests. A repeated measures analysis of variance (ANOVA) was conducted to assess mean differences across the three SORT trials. When a significant result was observed, pairwise comparisons with Bonferroni post-hoc adjustments were used to determine where differences existed. Composite Z-scores were created, and bivariate correlation analysis was conducted to compare Cooper’s Test results to obstacle course performances from the two previous years prior to the current investigation for participants participating in this pilot study. Similarly, composite z-scores of the SORT outcomes were created and compared, via bivariate correlation, to obstacle course performances for the same SWAT participants.

## 3. Results

Of the original 24 participants, 14 completed the study. The ten participants who did not complete all three trials cited time-related (family/work) commitments and financial restrictions for their inability to continue. No injuries were reported as a reason for non-completion. Descriptive data for SORT outcomes can be found in Table 1.

Among the SORT battery metrics, Pearson R values can be found in Table 2. All SORT events were significantly related to two or more other events except for the sled drag, which was only significantly correlated with weighted lunge performance (R = −0.726, *p* < 0.05). The strongest correlation presented was between the weighted push-ups and the isometric pull-up hold (R = 0.959, *p* < 0.01). The Yo-Yo presented the greatest number of strong (R > 0.7) correlations with other events, excluding the sled drag.

The ICC_2,1_ calculated for each component of the test battery reported significant repeatability over the three trials. The weighted lunge test presented moderate to exceptional repeatability with an ICC_2,1_ of 0.834 (95% CI [0.530, 0.945]) but the Yo-Yo test was highly repeatable at 0.980 (95% CI [0.939, 0.994]). The isometric pull-up hold ranged from good to excellent agreement across trials with an ICC_2,1_ of 0.918 (95% CI [0.761, 0.974]), whereas the weighted squat demonstrated poor to exceptional reliability with an ICC_2,1_ of 0.768 (95% CI [0.396, 0.923]). Lastly, the sled drag demonstrated exhibited poor to moderate agreement across trials with an ICC_2,1_ of 0.668 (95% CI [0.258, 0.878]), while the weighted push-up demonstrated moderate to excellent reliability with an ICC_2,1_ of 0.754 (95% CI [0.530, 0.945]).

The results of the COV across all three trials, across trials 1 and 2, and across trials 2 and 3 are presented in Table 3. The variability related to score dispersion was minimized between trials 2 and 3 for four of the six events. All COV greater than 10% during trials 1 and 2 decreased below 10% for trials 2 and 3; this included the weighted lunge, the weighted push-up and the weighted squat. The Yo-Yo had the smallest COV, at less than 1%, for all three trials, trials 1 and 2, and trials 2 and 3. COV did increase for the Yo-Yo and the isometric pull-up hold from trials 1 and 2 to trials 2 and 3. However, the increase was less than 1% for both. All tests’ COVs were below 10% when assessing trials 2 and 3. Thus, the SORT events have very little dispersion around the mean after two familiarization trials.

There were no significant differences in performance across all three repeated measures ANOVA trials for the weighted lunges, isometric pull-up hold, weighted squats, sled drag, or Yo-Yo. Weighted push-ups were the only significantly different SORT event across the trials (*p* = 0.002). Pairwise comparisons revealed push-ups to be significantly different between trials 1 and 2 (34.5 ± 10.2 reps vs. 40.3 ± 9.9 reps, *p* = 0.005) and trials 1 and 3 (34.5 ± 10.2 reps vs. 41.7 ± 9.5 reps, *p* = 0.015). Trials 2 and 3 were not significantly different from one another. Composite Z-scores from the two preceding years for the Cooper’s Test were significantly, strongly correlated with the obstacle course results of those same years, with R = −0.921 and R = −0.867, respectively (*p* = 0.003 and *p* < 0.001). SORT composite z-scores from trials 2 and 3 were also significantly and strongly correlated (R = −0.805 (*p* = 0.014) with obstacle course results collected two weeks prior to the present study. Participants completed the obstacle course in an average time of 177.5 ± 30.6 s.

**Figure 2:**
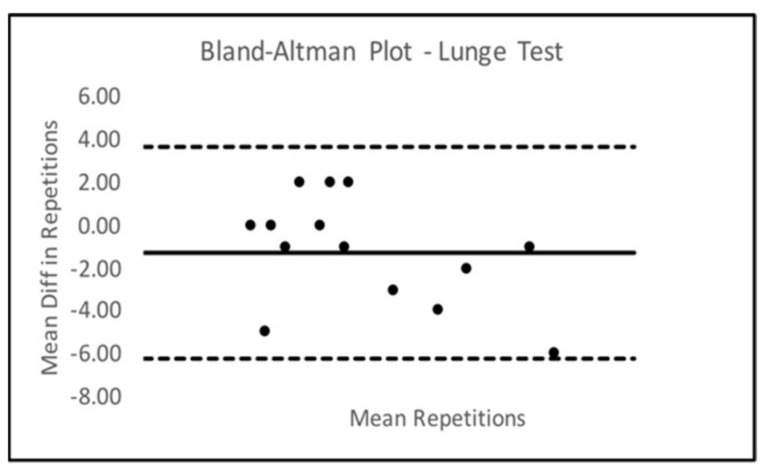
Bland–Altman Plot—Lunge.

**Figure 3:**
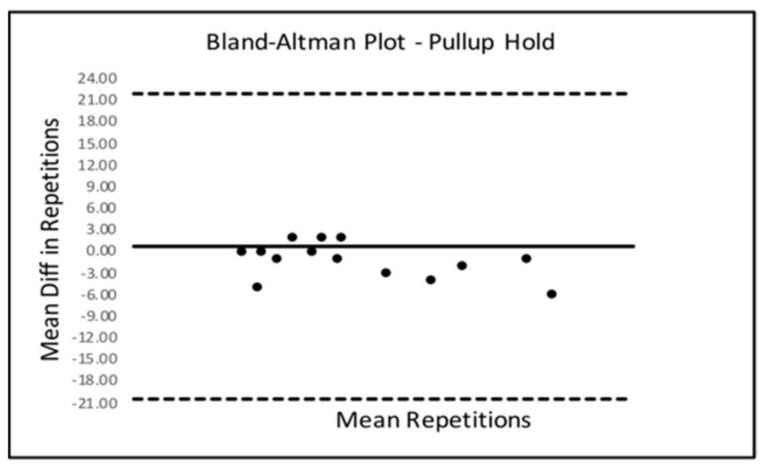
Bland–Altman Plot—Hold.

**Figure 4:**
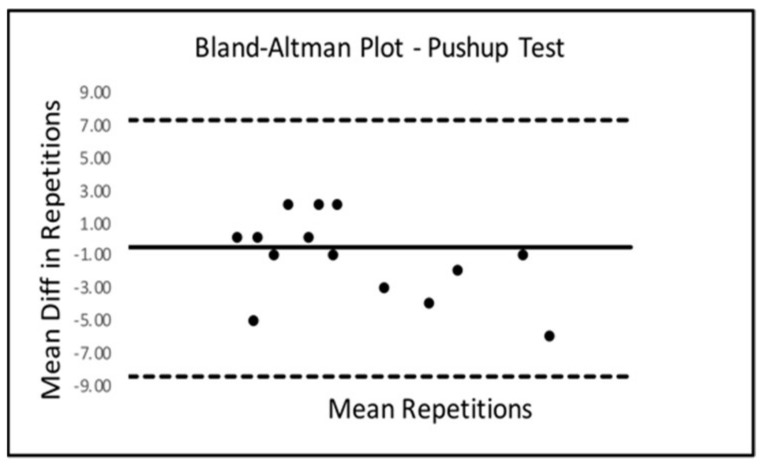
Bland–Altman Plot—Pushup.

**Figure 5:**
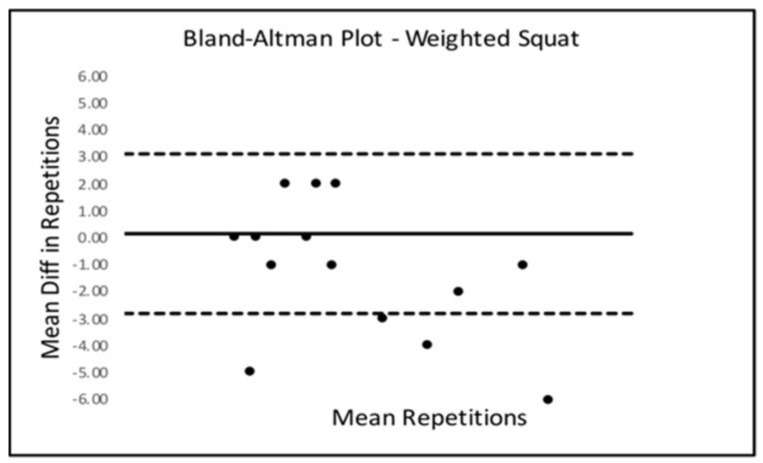
Bland–Altman Plot—Squat.

**Figure 6:**
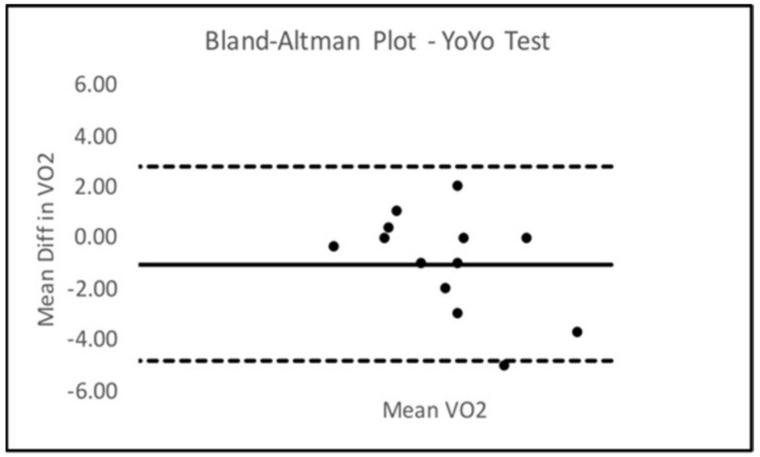
Bland–Altman Plot YoYo IR.

Bland–Altman Limits Of Agreement (LOA) plots were created to compare trials 2 and 3 for all six SORT battery tests. Only the sled drag event presented with a statistically significant mean difference from zero. The Bland–Altman LOA plots for all SORT events can be seen below in Figure 2, Figure 3, Figure 4, Figure 5, Figure 6 (excluding sled drag).

As demonstrated in the plots above, all tests appeared to show a dispersion of scores that remained within the upper and lower limits of agreement in each plot except for the weighted squat where scores seemed to depart agreement, possibly demonstrating some bias in performance across trials 2 and 3 for the squat event.

## 4. Discussion

The purpose of this study was to examine the test–retest reliability of the SORT battery and its appropriateness for use in SWAT populations. Additionally, the purpose was to compare the SORT to a previously used fitness test (Cooper’s Test) commonly used in LEOs and evaluate both against a criterion measure of SWAT obstacle course performance. The main findings were that most of the SORT events were significantly and strongly correlated with one another, all events demonstrated repeatability over three trials, the variability in score dispersion was minimized between trials 2 and 3, and both the SORT and Cooper’s Test were significantly and strongly correlated to obstacle course performance.

Correlations between SORT events were analyzed to establish which events were highly related to each other to better define common physical traits among the SWAT operators. Most SORT events were significantly and strongly correlated to one another. The sled drag, however, was the only event that presented just one significant correlation, R = −0.726, when compared with the weighted lunge. Both the sled drag and the weighted lunge require the SWAT operators to locomote while being under a load. Additionally, these two events are both unilateral in nature and require that the operators produce force primarily one leg at a time. This may account for why these two events were so strongly correlated. The weighted squat was unexpectedly not correlated with the sled drag, despite being performed under a load and taxing lower body musculature. This may be since the weighted squat tests maximal repetitions in 120 s and incorporates a pause component, where muscular endurance would be optimal, whereas the sled drag lasts, on average, 9.4 ± 2.6 s, and tests anaerobic power. Due to weak and insignificant correlations with other events, the sled drag may be removed from the SORT battery as it is correlated with the lunge and may be testing some of the same capacities. Additionally, the sled drag is a typical component of most SWAT obstacle courses and incorporating it in the SORT may be redundant and cause unnecessary fatigue during testing. An anaerobic power event, such as a loaded standing broad jump, may be an adequate replacement as this jumping event has been positively correlated with sled drag performance and may produce less fatigue [18].

The Yo-Yo, and similar shuttle style tests, have been used in other studies to assess cardiovascular fitness in tactical populations and has proved a valid and reliable metric [17,19,20]. SWAT operator performance on the Yo-Yo, therefore, is indicative of their cardiovascular fitness. The Yo-Yo consistently presented strong correlations (R > 0.7) with all other events, except the sled drag. Previous studies have drawn correlations between cardiovascular fitness and performance on occupationally specific tasks. Thomas and colleagues concluded that VO_2peak_ was negatively correlated to time to complete a tactical task while loaded [12]. That is, higher VO_2peak_ was correlated with decreased fatigue while completing the occupationally specific task. Cardiovascular fitness has been suggested to enhance recovery from high intensity intermittent exercise [21]. Thus, better performance in the Yo-Yo would correlate with enhanced recovery between the SORT metrics as well as reduced fatigue from load carriage.

The SORT battery metrics presented repeatability over the course of three trials. With all ICCs exceeding 0.7, the SORT battery is a reliable test for use in SWAT Operators. However, the SORT may require a few familiarization trials. Based on COV, the variability of score dispersion around the mean was decreased between trials 2 and 3 compared to trials 1 and 2 for most of the SORT events. Weighted push-up outcomes were significantly better in trials 2 and 3, compared to trial 1. While no other metrics were significantly different between trials, based on the trend that COV decreased upon further trials, at least two familiarizations are recommended to combat the effects of learning. The SORT may be a more reliable test battery than a fitness test with set standards, such as Cooper’s Test, because minimum standards may present little incentive for maximum effort.

Cooper’s Test and the SORT were both significantly and strongly correlated to a criterion measure; performance on an obstacle course that was designed to test qualification to become a SWAT operator. Cooper’s Test was hypothesized to have an insignificant correlation with obstacle course outcomes. Nevertheless, the two measures were strongly correlated. Therefore, Cooper’s Test is a better indicator of SWAT operator performance on a test with criterion validity than originally believed. Performance on a fitness test similar to Cooper’s Test (tests of local muscular endurance, a 201 m sprint, and 2.4 km run) was correlated with performance on a work battery test in law enforcement recruits [4]. Similarly, tests of relative VO_2peak_ and local muscular endurance presented significant correlations with simulated foot chase tasks in LEOs [22]. However, Cooper’s Test and derivatives may fail to assess a key component of SWAT operator readiness: performance under loaded conditions.

The SORT presented a significant and strong correlation with obstacle course performance. This correlation, however, was weaker than that of Cooper’s Test (R = −0.737 vs. R = −0.921 and R = −0.867). This may be due to the obstacle course being completed in an unloaded condition, like Cooper’s Test, rather than a loaded condition similar to the SORT and typical operational duties. Nevertheless, the SORT is correlated with performance on a criterion measure and was able to reliably assess SWAT operators’ performance on tasks that the literature has shown to be essential. SWAT operators often perform explosive movements after remaining in a tactical position for an extended period of time [11]. Thus, the weighted lunge matrix and weighted paused squat are indicative of the ability of a SWAT operator to function under their PPE load and remain mobile while on the job. SWAT operators must also carry a ballistic shield, requiring the isometric use of the upper body [11]. Accordingly, the isometric pull-up hold may be indicative of a SWAT operator’s ability to perform this task as well as defend themselves in a hand-to-hand combat situation.

While other fitness tests have incorporated a push-up event, adding an 18.2 kg weighted vest to the event more closely resembles the requirements of a SWAT operator while pushing oneself over an obstacle or breaching a door while wearing their PPE, or perhaps even pushing themselves up from a prone firing position [10]. The use of a sled drag event may be redundant due to the specific obstacle course this SWAT unit employs. Regardless, lifting something from the floor/knee height greater than 150 lbs was the most common task in surveyed SWAT operators and dragging a fully equipped casualty to safety was also common [6,10]. Keeping the sled drag as a SORT metric may be indicative of this ability and crucial for SWAT units that do not incorporate a sled drag, or “Man Down” event, in their fitness test. Lastly, cardiovascular fitness has been cited as a key fitness measure in SWAT operators and has been correlated with occupational performance numerous times [10,22,23,24,25]. Therefore, the Yo-Yo, a valid and reliable test to predict VO_2max_, is essential in its inclusion in the SORT.

Per the results of this study, the SORT is a valid and reliable measure of physical fitness in SWAT operators. Cooper’s Test is also presented as valid in comparison to a criterion measure. Thus, both tests may be predictive of obstacle course, and possibly occupational, performance. While these two tests may not be statistically different in their ability to determine occupational readiness per unloaded obstacle course performance, the SORT may better assess the key components of physical fitness, such as muscular strength, mobility and agility, that Cooper’s Test does not. Additionally, the SORT requires SWAT operators to test three of the six events under a loaded condition. This may be more representative of operational requirements than Cooper’s Test. The SORT, therefore, may be able to better expose weaknesses in SWAT operators compared to Cooper’s Test, which only addresses unloaded local muscular endurance, speed, and cardiovascular fitness.

Due to the large attrition rate in this study (24 operators began the study and only 14 finished due to previously mentioned concerns), the small sample size is a limitation. Future studies should utilize a larger SWAT population composed of both part-time and full-time operators from suburban and urban units. Additionally, a limitation was the lack of our ability to directly compare obstacle course results from the most recent test to Cooper’s Test results. Due to the part-time nature of the SWAT operators and significant time spent volunteering on the three SORT trials and obstacle course, Cooper’s Test was not assessed for the current population. However, the current SWAT command had already discontinued the use of Cooper’s Test due to its inability to accurately represent all fitness components necessary in SWAT operators. Establishing the validity and reliability of the SORT was paramount in this investigation. However, future studies should examine whether the SORT can be performed with in-house equipment, such as PPE and a fully loaded operator, instead of requiring the purchase of weighted vests and sleds.

## 5. Conclusions

The SORT battery could be used as a valid and reliable testing measure in SWAT populations to assess occupationally specific fitness components. The SORT was strongly correlated with performance on a criterion measure (SWAT obstacle course) and assesses fitness components that typical law enforcement fitness tests fail to measure. Because SWAT operators have additional physical demands compared to LEOs, a fitness test that incorporates a load carriage component is of utmost importance. To our knowledge, this is the first field-based physical fitness test to assess strength and mobility in loaded SWAT operators while also including tests of muscular endurance, anaerobic power and cardiovascular fitness. Because loaded conditions are so detrimental to performance, identifying these weaknesses before an operator is on the job is integral to mission safety and success [12,13]. Thus, training programs and fitness tests should be tailored towards SWAT operators’ unique requirements. The SORT battery appears to be a valid and reliable test for use in this population.

## Figures and Tables

**Table 1:** Descriptive data for the SORT battery in part-time SWAT operators (*n* = 14).

Variables	Mean ± SD
Lunge (repetitions)	20.9 ± 8.9
Push-Up (repetitions)	37.6 ± 10.0
Pull-Up (seconds)	47.1 ± 18.4
Squat (repetitions)	13.5 ± 4.4
Sled Drag (seconds)	9.4 ± 2.6
Yo-Yo (mL/kg/min)	41.7 ± 0.4

**Table 2:** Pearson Correlation Coefficient values between SORT events (*n* = 14).

	Lunge	Push-Up	Pull-Up	Squat	Sled	Yo-Yo
Lunge	1.00	0.638 *	0.601	0.802 **	−0.726 *	0.894 **
Push-Up	0.639 *	1.00	0.959 **	0.769 **	−0.041	0.790 **
Pull-Up	0.601	0.959 **	1.00	0.684 *	−0.024	0.769 **
Squat	0.802 **	0.769 **	0.684 *	1.00	−0.363	−0.803 **
Sled	−0.726 *	−0.041	−0.024	−0.363	1.00	−0.537
Yo-Yo	0.894 **	0.790**	0.769 **	0.803 **	−0.537	1.00

* indicates statistical significance of *p* ≤ 0.05. ** indicates statistical significance of *p* ≤ 0.01.

**Table 3:** Coefficient of variation across SORT battery trials (*n* = 14).

	Trials 1, 2, 3	Trials 1 & 2	Trials 2 & 3
Lunge	10.18%	11.16%	7.72%
Push-Up	12.75%	13.26%	8.44%
Pull-Up	9.67%	8.02%	8.97%
Squat	11.22%	13.11%	6.71%
Sled Drag	10.84%	9.48%	5.42%
Yo-Yo	0.94%	0.77%	0.95%
